# Personalization of AI Using Personal Foundation Models Can Lead to More Precise Digital Therapeutics

**DOI:** 10.2196/55530

**Published:** 2025-08-21

**Authors:** Peter Washington

**Affiliations:** 1 Department of Medicine Division of Clinical Informatics and Digital Transformation University of California, San Francisco San Francisco, CA United States

**Keywords:** precision health, deep learning, self-supervised learning, patient generated health data, digital therapeutics, therapeutic, digital health solution, machine learning, artificial intelligence, model, patient data, health outcome, deep learning model, perspective

## Abstract

Digital health interventions often use machine learning (ML) models to make predictions of repeated adverse health events. For example, models may be used to analyze patient data to identify patterns that can anticipate the likelihood of disease exacerbations, enabling timely interventions and personalized treatment plans. However, many digital health applications require the prediction of highly heterogeneous and nuanced health events. The cross-subject variability of these events makes traditional ML approaches, where a single generalized model is trained to classify a particular condition, unlikely to generalize to patients outside of the training set. A natural solution is to train a separate model for each individual or subgroup, essentially overfitting the model to the unique characteristics of the individual without negatively overfitting in terms of the desired prediction task. Such an approach has traditionally required extensive data labels from each individual, a reality that has rendered personalized ML infeasible for precision health care. The recent popularization of self-supervised learning, however, provides a solution to this issue: by pretraining deep learning models on the vast array of unlabeled data streams arising from patient-generated health data, personalized models can be fine-tuned to predict the health outcome of interest with fewer labels than purely supervised approaches, making personalization of deep learning models much more achievable from a practical perspective. This perspective describes the current state-of-the-art in both self-supervised learning and ML personalization for health care as well as growing efforts to combine these two ideas by conducting self-supervised pretraining on an individual’s data. However, there are practical challenges that must be addressed in order to fully realize this potential, such as human-computer interaction innovations to ensure consistent labeling practices within a single participant.

## Introduction

In recent years, the intersection of consumer digital health and machine learning (ML) has emerged to enable ML-powered digital therapeutics, which have been developed in areas such as interventions for substance use [1-4]; technologies for managing mental health conditions such as anxiety, stress, and depression [5-8]; and autism therapeutics using Google Glass [9,10]. The models powering these digital therapies typically analyze large streams of an individual patient’s data in order to anticipate adverse health events or actionable patient-reported outcomes. However, a significant computational challenge arises when dealing with the prediction of nuanced and subjective health events that are typically self-reported by participants in the form of patient-reported outcomes, such as mental health states like stress and anxiety. For such prediction targets, the cross-subject variability poses an obstacle for traditional ML approaches, as one participant’s label of “moderately stressed” might be another participant’s “lightly stressed”.

Conventional ML methodologies typically involve training a single generalized model to classify a specific condition [11], such as for diagnostic or screening purposes. However, attempting to apply a universal model often leads to poor generalization to individuals and health systems that were not represented in the training data. An alternative solution involves training separate models for each individual or subgroup, tailoring the model to the unique characteristics of the patient. However, this approach would traditionally demand extensive labeled data from each participant, a requirement that has historically hindered the feasibility of personalized ML applications in precision health care.

The relatively recent advent of self-supervised learning (SSL), made popular in the context of pretraining large language models like ChatGPT (OpenAI), has enabled a transformative solution to address the challenges associated with personalized ML in health care [12-14]. SSL is a machine learning paradigm in which a model is trained to understand and represent the underlying structure of its input data without relying on externally provided labels. By pretraining deep learning models on vast amounts of unlabeled data streams derived from patient-generated health data to understand the baseline temporal dynamics of the data stream without a single label, SSL provides a means to fine-tune personalized models with significantly fewer labeled data points than when using traditionally supervised learning. This relatively new paradigm opens new avenues for making ML personalization in health care more practical, thereby overcoming one of the major hurdles that has historically impeded progress in this area.

This perspective explores the integration of SSL and personalization in scenarios where there are large unlabeled data streams generated per patient, focusing in particular on the potential of personalized SSL to improve the performance of digital therapeutics that provide some sort of digital therapy or digital intervention when a prediction about the participant in question is made by an ML model.

## Personalized Models in Health Care

**Figure 1:**
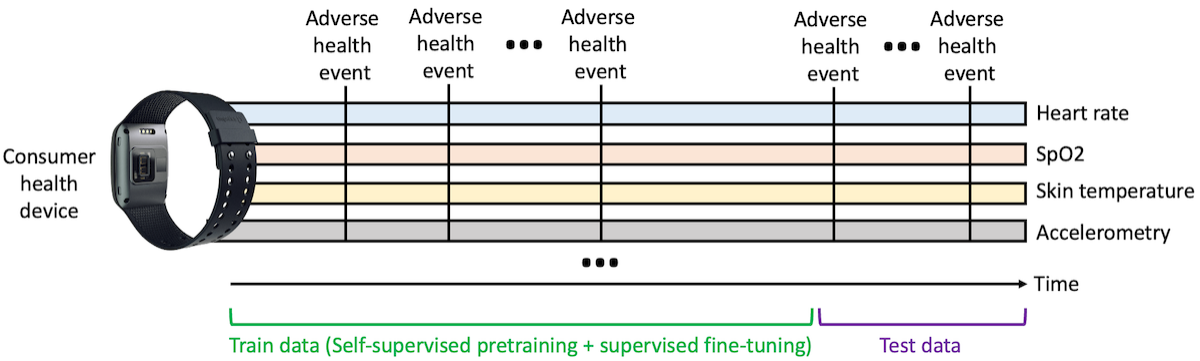
In many biomedical domains, there exist massive unlabeled data streams with sparse annotations of the health event of interest. In personalized self-supervised learning, we can pretrain on data coming earlier from the participant and then fine-tune on an ideally small number of patient-provided labels. Evaluation then occurs later temporally. HR: heart rate; SpO2: oxygen saturation.

Traditional ML methodologies, which often rely on a one-size-fits-all model, face substantial challenges when confronted with the diverse and nuanced nature of health outcomes. The need for personalized models that cater to individual characteristics has led to a paradigm where a single ML model is trained on data streams coming from a single user and evaluated on future data coming from that same user (Figure 1).

Several examples of personalized ML models for health care have been published in the past decade. Zhang et al [15] developed Patient2Vec, a representation learning approach for longitudinal electronic health record data used to predict clinical events into the future. Luu et al [16] trained a generalized model that was then fine-tuned to predict step count in a personalized manner, achieving 98%-99% accuracy in the personalized case and 96%-99% accuracy with the generalized models. Li et al [17] compared a personalized model for stress prediction against 2 baselines, subject-inclusive and subject-exclusive generalized models, finding that the personalized models significantly outperformed both sets of generalized models. This finding indicates that personalization using only an individual’s data outperforms personalization when combining the personal data with data from other users, at least for highly heterogeneous outcomes such as affective computing.

Federated learning, where distributed local models are trained and sent to a central global server for weight aggregation, is naturally connected to the idea of personalized ML. Each “local” model is, by definition, a personalized model. Federated learning has been successfully applied to certain health care settings. For example, Rudovic et al [18] developed a personalized federated learning approach for pain estimation from face images where clients train models using local data, aggregate the model weights in a central server, and then send the global model back to the clients for fine-tuning. This federated learning approach enables the classification of a traditionally difficult classification task due to its inherent subjectivity and heterogeneity between individuals, namely, pain estimation using computer vision.

Traditional applications of personalized ML apply to scenarios where there are vast amounts of data labels per patient. Unfortunately, this situation is often unattainable. In contexts where the data labels pertain to patient-generated health data, it is especially infeasible to collect many labels. To address this practical issue with traditional personalized ML, this perspective explores the idea of performing SSL on an individual’s unlabeled data streams to create a personalized foundation model.

## Personalized Foundation Models: Combining Personalization With Self-Supervised Learning

SSL holds great promise to improve the performance of ML models in health care [19], broadly speaking. SSL involves leveraging the inherent information within the data itself to create supervisory signals for training. SSL has been traditionally applied to large datasets containing data from a broad array of patients. In passive data generation contexts, however, such as when patients wear a monitor that continuously collects biosignals, it can be productive to run SSL separately for each patient, as each patient has a large amount of data sampled several times a second. These separate pretraining procedures per patient can result in a “personal foundation model.” Because foundation models can learn using much less data than would have been required if no SSL took place, the personal foundation models can enable learning of complex health outcomes where the supervisory signal drastically varies across patients.

**Figure 2:**
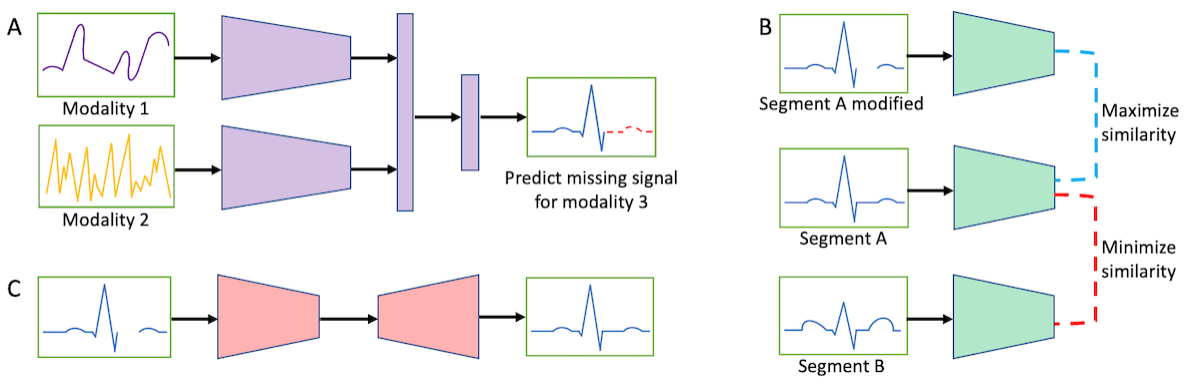
Examples of self-supervised learning approaches for longitudinal time series data. (A) An inherently multimodal approach is to predict the missing portion of a signal given the values of signals from separate data modalities. (B) Another approach is to perform contrastive learning on the signals by training a network to maximize similarity between a data point and an augmented version of that data point while minimizing similarity between that data point and a separate data point. (C) A third possible strategy is to predict the missing portion of a signal using a masked autoencoder or similar model.

SSL for personalization of longitudinal time series data for health care can be achieved through a variety of adaptations of popular SSL pretraining strategies (Figure 2). An inherently multimodal approach is to predict the missing portion of a signal given the values of signals from separate data modalities (Figure 2A) [20], treating the prediction as a multiple-output regression task [21]. Another approach is to perform contrastive learning algorithms such as SimCLR [22] on the signals to maximize representational similarity between augmented versions of the same time period while minimizing similarity between 2 distinct time windows (Figure 2B) [23,24]. More sophisticated generative approaches, such as masked autoencoders [25] and latent masking [26], can also be used to predict masked portions of input signals (Figure 2C), including in a multimodal manner [27].

Personalized modeling combined with SSL has recently enabled the successful prediction of traditionally heterogeneous and subjective health outcomes. For example, Li and Sano [28] used unsupervised representation learning to predict outcomes related to wellbeing, such as mood and stress. Li et al [29] computed personalized brain function networks from functional magnetic resonance imaging using SSL. Spathis et al [30] used SSL to learn user-specific representations of wearable data streams and demonstrated that these personalized representations can be fine-tuned to a variety of downstream tasks.

One important consideration is that increases in model performance might be due to either the personalization aspect or the SSL aspect. SSL without personalization has been repeatedly documented to improve ML model performance [31-34]. Thus, it is important to systematically isolate both conditions in isolation as baselines to determine the true contribution of each component.

Another caveat to personalized SSL is that within-subject consistency in labeling is crucial, and initial studies have found that improvement gains observed using personalized SSL require consistency in data labeling within a user. For example, Islam and Washington [35,36] applied personalized multimodal SSL to the Wearable Stress and Affect Detection dataset [37], observing significant improvements in model performance when compared to a baseline model using identical data without self-supervised pretraining. By contrast, Eom et al [38] evaluated a multimodal dataset collected by Hosseini et al [39] consisting of wearable biosensors measured from nurses working during the COVID-19 outbreak. Eom et al [38] did not observe increased performance on average when using personalized models pretrained on each individual’s data compared to baseline models, likely due to particularly noisy and irregular data collection procedures arising from nurses providing data during a stressful event. This highlights the importance of using datasets that have consistent labeling within a participant in order to make personalized SSL actually work.

## Future Opportunities

Applications of personalized SSL to recurrent health predictions have been successful thus far under clean data scenarios. By harnessing the power of SSL, these applications have demonstrated the ability to glean intricate patterns and dependencies within longitudinal health data. As advancements continue in this burgeoning field, the promise of enhanced precision, early intervention, and improved overall health outcomes appear increasingly attainable for health domains and datasets that are traditionally “challenging” due to their inherent subjectivity, heterogeneity, and complexity.

Despite the initial successes described here, there are likely myriad digital health applications that have yet to be realized because they were not previously feasible prior to the advent of SSL. For example, recent advances in personalized SSL for emotion recognition [40] have the potential to improve the efficiency of the personalization of artificial intelligence–powered digital therapeutics for children with autism [41,42]. While the state-of-the-art of emotion recognition models hovers around 70% accuracy [43], previous emotion personalization efforts without self-supervision were able to achieve strong performances [44]. It is likely that further improvements with fewer labels will be possible with personalized SSL. This approach has yet to be applied to digital therapeutics more broadly, and this gap suggests the possibility of more precise digital therapeutics in the coming years.

## Ongoing Challenges

Personalized SSL studies can often be framed as several independent N=1 studies, where each study and corresponding model consists of training, validation, and testing data that all come from a single user. Such studies must be careful about overfitting across 2 dimensions: within subjects and between subjects. While between-subject overfitting, or overfitting to some patients while failing to generalize to other patients, is often discussed, discussions and evaluations of overfitting within a subject appear relatively sparse in the literature. Future work should explore overfitting in this temporal dimension more thoroughly.

Another ill-studied area is the intersection of performance discrepancies and personalization. Personalization of models should, in theory, lead to a reduction in ML performance discrepancies across groups. The capability of model personalization to reduce these discrepancies has yet to be thoroughly studied. However, it is plausible that personalized models could still propagate existing performance gaps across groups if the underlying data remains skewed or if the personalization process disproportionately benefits certain groups [45]. A thorough understanding of this will require rigorous evaluation across a wide range of populations.

Another key challenge of personalized foundation models is that individuals change over time. As an extreme example to illustrate the point, a personalized model that was trained on an individual during their youth may be irrelevant during their 30s. The paradigm of continual (or online) learning, or the continual retraining of models as new data become available, can offer a solution. By allowing models to adapt incrementally, continuous learning can ensure that they evolve alongside the user, capturing shifts in behavior, preferences, and needs over time. Possible approaches can include incremental fine-tuning [46-48], where the model is periodically retrained on newly available data while retaining previously learned weights; experience replay [49,50], where a subset of past data is stored and combined with new data during model updates; and meta-learning [51,52], where the model learns how to quickly adapt to new data by leveraging prior knowledge, making it efficient in learning new tasks from fewer examples.

A final critical challenge is addressing human factors that influence the quality, consistency, and usability of patient-generated data in personalized SSL pipelines. As Slade et al [53,54] highlight, participants often encounter both technical and behavioral barriers during data collection, including device discomfort, app usability issues, and low perceived relevance of labeling tasks. These factors can lead to sporadic participant engagement, mislabeled or missing data, and dropout, ultimately undermining the effectiveness of models that rely on temporal consistency and high-volume personal data streams. Designing for human factors through mechanisms such as clearer feedback loops, improved incentives, and user-centered data collection interfaces will be essential to support robust protocol adherence leading to successful personalization.

## Conclusion

The training of personalized foundation models by learning from the vast unlabeled time series data that are often generated from patients can lead to ML applications in health care that expand beyond the traditional realm of diagnostics, such as adaptive and customized digital therapeutics. This area of research is relatively understudied in comparison to other aspects of ML-powered digital health, though it is likely that the advent and increasingly widespread application of SSL will lead to a proliferation of such applications.

## Abbreviations

ML: machine learning
SSL: self-supervised learning
